# DNA Stable-Isotope Probing Delineates Carbon Flows from Rice Residues into Soil Microbial Communities Depending on Fertilization

**DOI:** 10.1128/AEM.02151-19

**Published:** 2020-03-18

**Authors:** Yali Kong, Yakov Kuzyakov, Yang Ruan, Junwei Zhang, Tingting Wang, Min Wang, Shiwei Guo, Qirong Shen, Ning Ling

**Affiliations:** a Jiangsu Provincial Key Lab for Organic Solid Waste Utilization, Jiangsu Collaborative Innovation Center for Solid Organic Waste Resource Utilization, Nanjing Agricultural University, Nanjing, China; b Department of Soil Science of Temperate Ecosystems, Department of Agricultural Soil Science, University of Goettingen, Göttingen, Germany; c Agro-Technology Institute, RUDN University, Moscow, Russia; Michigan State University

**Keywords:** fertilization, ^13^C-labeled rice residue, DNA stable-isotope probing, high-throughput sequencing, active microbes

## Abstract

Decomposition of crop residues in soil is mediated by microorganisms whose activities vary with fertilization. The complexity of active microorganisms and their interactions utilizing residues is impossible to disentangle without isotope applications. Thus, ^13^C-labeled rice residues were employed, and DNA stable-isotope probing (DNA-SIP) combined with high-throughput sequencing was applied to identify microbes active in assimilating residue carbon (C). Manure addition strongly modified microbial community compositions involved in the C flow from rice residues. Relative abundances of the bacterial genus *Lysobacter* and fungal genus *Syncephalis* were increased, but abundances of the bacterial genus *Streptomyces* and fungal genus *Trichoderma* were decreased in soils receiving mineral fertilizers plus manure (NPKM) compared to levels in soils receiving only mineral fertilizers (NPK). Microbes involved in the flow of residue C formed a more complex network in NPKM than in NPK soils because of the necessity to decompose more diverse organic compounds. The fungal species (*Jugulospora rotula* and *Emericellopsis terricola* in NPK and NPKM soils, respectively) were identified as keystone species in the network and may significantly contribute to residue C decomposition. Most of the fungal genera in NPKM soils, especially *Chaetomium*, *Staphylotrichum, Penicillium*, and *Aspergillus*, responded faster to residue addition than those in NPK soils. This is connected with the changes in the composition of the rice residue during degradation and with fungal adaptation (abundance and activity) to continuous manure input. Our findings provide fundamental information about the roles of key microbial groups in residue decomposition and offer important cues on manipulating the soil microbiome for residue utilization and C sequestration in soil.

**IMPORTANCE** Identifying and understanding the active microbial communities and interactions involved in plant residue utilization are key questions to elucidate the transformation of soil organic matter (SOM) in agricultural ecosystems. Microbial community composition responds strongly to management, but little is known about specific microbial groups involved in plant residue utilization and, consequently, microbial functions under different methods of fertilization. We combined DNA stable-isotope (^13^C) probing and high-throughput sequencing to identify active fungal and bacterial groups degrading residues in soils after 3 years of mineral fertilization with and without manure. Manuring changed the active microbial composition and complexified microbial interactions involved in residue C flow. Most fungal genera, especially *Chaetomium*, *Staphylotrichum, Penicillium*, and *Aspergillus*, responded to residue addition faster in soils that historically had received manure. We generated a valuable library of microorganisms involved in plant residue utilization for future targeted research to exploit specific functions of microbial groups in organic matter utilization and C sequestration.

## Abstract (precis)

Identifying and understanding the active microbial communities and interactions involved in plant residue utilization are key questions to elucidate the transformation of soil organic matter (SOM) in agricultural ecosystems. Microbial community composition responds strongly to management, but little is known about specific microbial groups involved in plant residue utilization and, consequently, microbial functions under different methods of fertilization. We combined DNA stable-isotope (^13^C) probing and high-throughput sequencing to identify active fungal and bacterial groups degrading residues in soils after 3 years of mineral fertilization with and without manure. Manuring changed the active microbial composition and complexified microbial interactions involved in residue C flow. Most fungal genera, especially *Chaetomium*, *Staphylotrichum, Penicillium*, and *Aspergillus*, responded to residue addition faster in soils that historically had received manure. We generated a valuable library of microorganisms involved in plant residue utilization for future targeted research to exploit specific functions of microbial groups in organic matter utilization and C sequestration.

## INTRODUCTION

The importance of microbial residues for soil organic matter (SOM) formation has been recognized for over 2 decades (1, 2), indicating the crucial roles of microbes in sustainability and soil fertility. Therefore, dominant microbes involved in decomposition play important roles for biologically driven carbon (C) in C flows. The development of molecular biology techniques has greatly advanced our understanding of microorganisms that mediate the decomposition of plant residue in soil (3–6). To sequester more C in soil during plant residue decomposition, we need to consider the C pathways from the residue into the soil organic matter through microbial processing (7). Therefore, precise identification of the microbial groups involved in C utilization and sequestration is the key to exploring the functional roles of microbes in improving soil fertility.

Approaches using stable-isotope probing (SIP) provide novel insights into C flows in soil microbial communities. The identity of microorganisms has been linked with their activities and functions using SIP, for example, in tracing the fate of low-molecular-weight organic substrates, such as glucose (8–12), methanol (13, 14), and propionate (15). A few studies considering more complex organic compounds focused on root exudates (16–20) or high-molecular-weight organic substrates, such as cellulose (21, 22). A number of studies used SIP to characterize specific microbial groups utilizing plant residues differing in qualities or types, but all of these studies concentrated on only a single soil condition (23–29). Limited information is available to address whether the active microbiome involved in residue decomposition will be affected by specific fertilization practices although the total soil microbial community has been widely reported to be changed (30, 31).

In agricultural soils, changes in the C availability due to fertilization, such as mineral or organic fertilization, always alter microbial community structure (32, 33), which further leads to alterations in the enzyme activities related to C and N cycling (34–38). The application of organic fertilizers (i.e., cattle manure and compost) consistently resulted in higher levels of cultivable microorganisms in soil and of enzyme activities and thus can be used as an environmentally friendly and rapid measure for restoring degraded cropland (39). The organic amendments also significantly impact the decomposition rate of crop residues (40). So far, no attempt has been made to evaluate the effects of mineral and organic fertilizers on microbial groups decomposing plant residues. Considering that the degradation of plant residues is a microbe-mediated process and that fertilization selects for specifically adapted microbial communities, it is reasonable to propose that distinct active microbial communities responding to residue decomposition can exist in soils experiencing contrasting fertilization regimes. Mineral, in contrast to organic, inputs alter the general balance of soil stoichiometry, thereby resulting in adaptative selection of a resident population of oligotrophic taxa (high C use efficiency, slow growing) instead of more copiotrophic taxa (low C use efficiency, fast growing). Wang et al. (41) reported that the application of organic fertilizers increased the abundance of generally copiotrophic bacterial groups, while application of mineral fertilizers increased the abundance of oligotrophic groups. Thus, we hypothesized that there will be a shift in dominance and response strategies of specific microbial groups involved in residue assimilation in soils after manuring compared to these strategies after only mineral fertilization. To clarify this, soils were collected from a field experiment with different 3-year fertilization regimes, including fertilization with solely mineral fertilizers (NPK) and fertilization with mineral fertilizers plus manure (NPKM), and a microcosm experiment with ^13^C-labeled rice residues was conducted coupled with DNA-SIP technology.

## RESULTS

Rice residue (0.05 g rice residue/g dry weight soil [dws]) was employed to conduct microcosm experiments. A similar amount of about 0.04 to 0.05 g of residue/g soil (dry weight) was also employed in previous SIP studies investigating microbial communities involved in residue assimilation (26–28, 42). The choice of an incubation duration of 60 days was based on a previous study showing that the cumulative CO_2_ efflux at the first 60 days was nearly 70% of the total 160 days’ cumulative CO_2_ efflux after the addition of rice residues at 25°C (43). Our data (see Fig. S1 in the supplemental material) also showed that the CO_2_ efflux rates were relatively stable during 30 to 60 days after the residue amendment. In addition, microbial communities are mainly affected by the addition of residue or carbon substrates (such as glucose or cellulose) during the first 7 to 31 days of incubation (26, 28, 44, 45). In this study, due to the disturbance of soil during the rice residue amendment, soils were allowed to equilibrate for 7 days prior to the first sampling. Thus, days 7, 15, 30, and 60 after rice residue addition were selected as sampling points.

### Microbial communities utilizing residue-derived C.

The CO_2_ efflux rates and the abundances of total soil bacteria and fungi were significantly increased after the rice residue addition (Fig. S1). The dynamics of microbial abundances were similar in soils with and without manure addition (NPK plus residue and NPKM plus residue) (Fig. S1). According to the relative abundance distribution of the fungal (internal transcribed spacer [ITS]) and bacterial (16S) genes in the density gradient (Fig. S2), the fungal and bacterial DNAs from microorganisms utilizing ^13^C-labeled residues were clearly separated in the density gradient from 1.720 to 1.730 g ml^−1^ and from 1.730 to 1.740 g ml^−1^, respectively. The ^13^C-labeled DNA from the NPK plus ^13^C-labeled residue and NPKM plus ^13^C-labeled residue soil samples and the corresponding fractions from the NPK plus ^12^C-labeled residue and NPKM plus ^12^C-labeled residue soils were isolated for use in sequencing of the fungal ITS1 and bacterial 16S rRNA genes (V4-V5 regions).

**FIG 1:**
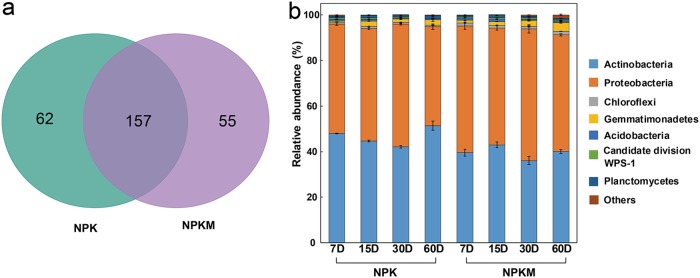
Effects of fertilization on the number (a) and relative abundance at the phylum level (b) of the ^13^C-labeled bacterial OTUs. Sampling was performed on the indicated days (D; *x* axis) after the addition of the rice residues. NPK, soil treated with mineral-only fertilizers; NPKM, soil treated with mineral fertilizers plus manure.

The operational taxonomic units (OTUs) were identified as being significantly labeled based on log_2_ fold change analysis. In total, of 219 and 212 bacterial OTUs were ^13^C labeled in the NPK and NPKM soils, respectively (Fig. 1a), whereas 157 of the OTUs were shared in soils with NPK and NPKM fertilization. *Proteobacteria* accounted for 46.5% and 39.6% of the total ^13^C-labeled bacterial communities in the NPK and NPKM soils, respectively, while *Actinobacteria* accounted for 48.6% and 54% (Fig. 1b). This indicates that *Proteobacteria* and *Actinobacteria* dominated the rice residue decomposition. The relative abundance of *Proteobacteria* was higher in NPKM soil than in NPK soil, whereas the relative abundance of *Actinobacteria* had the opposite trend. Besides these phyla, five other phyla, namely, *Chloroflexi*, *Gemmatimonadetes*, *Acidobacteria*, *Planctomycetes*, and candidate division WPS-1, also participated in residue decomposition (Fig. 1b).

**FIG 2:**
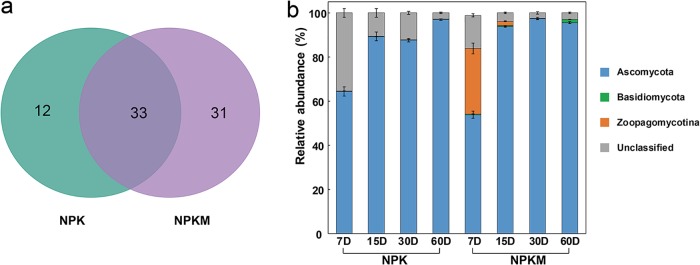
Effects of fertilization regimes on the number (a) and relative abundance at the phylum level (b) of the ^13^C-labeled fungal OTUs. Sampling was performed on the indicated days (D; *x* axis) after the addition of the rice residues. NPK, soil treated with mineral-only fertilizers; NPKM, soil treated with mineral fertilizers plus manure.

The number of ^13^C-enriched fungal OTUs was less than that of bacterial OTUs, with 45 fungal OTUs ^13^C labeled in NPK soil and 64 ^13^C labeled in NPKM soil (Fig. 2a). In NPK soil, residue-derived C was assimilated mainly by *Ascomycota* and *Basidiomycota* (Fig. 2b). The relative abundance of ^13^C-labeled *Ascomycota* gradually increased with time, from 64.4% on day 7 to 97% on day 60. In NPKM soil, in addition to the members of *Ascomycota* and *Basidiomycota*, *Zoopagomycotina* was also responsible for the residue C decomposition and utilization. The relative abundance of *Zoopagomycotina* gradually decreased from 29.6% on day 7 to 1.92% on day 15, and it was absent on day 30. In contrast, the relative abundance of *Ascomycota* gradually increased from 53.8% on day 7 to 95.6% on day 60 (Fig. 2b).

**FIG 3:**
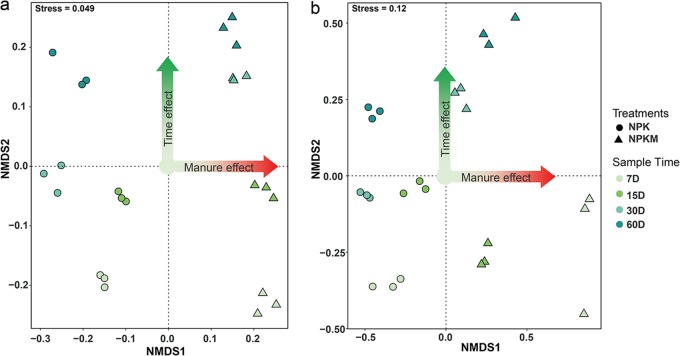
NMDS analyses of the ^13^C-labeled bacterial communities (a) and fungal communities (b) involved in the utilization of rice residues. Sampling was performed on the indicated days (D) after the addition of the rice residues. NPK, soil treated with mineral-only fertilizers; NPKM, soil treated with mineral fertilizers plus manure.

Nonmetric multidimensional scaling (NMDS) ordination of the ^13^C-labeled bacterial (stress value of 0.049) and fungal (stress value of 0.12) communities varied between the NPK and NPKM treatments on the horizontal axis and also clustered according to the different times on the vertical axis (Fig. 3). In addition, adonis analysis also showed that the ^13^C-labeled bacterial and fungal communities were significantly changed by fertilization and sampling time (Table S1). Therefore, in the following analyses, we focused mainly on the microbes that played dominant roles in the C flow depending on fertilization and on their dynamics based on the analysis of response strategies.

### Dominant microbes involved in the flow of residue-derived C.

**FIG 4:**
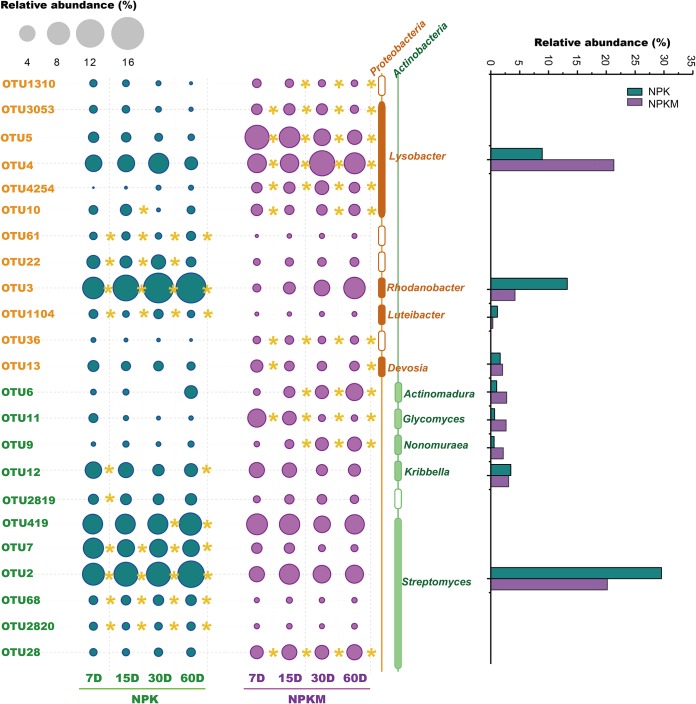
Dominant OTUs (relative abundance higher than 1%) in the bacterial communities involved in the C flows from rice residues. The size of the circle represents the relative abundance of the OTU. The color of the circle represents the fertilization treatment, as indicated. Sampling was performed on the indicated days (D) after the addition of the rice residues. The yellow asterisk (*) to right of a circle indicates that the relative abundance with the respective treatment was higher than that with the other treatment. Taxa are indicated to the right of the plot. The histogram in the right panel represents the average relative abundances of the dominant genera at the four sampling time points.

OTUs with relative abundances higher than 1% were identified as the dominant microbes. The dominant bacterial OTUs were mainly *Proteobacteria* and *Actinobacteria*, and the annotated OTUs belonged mainly to *Lysobacter*, *Devosia*, *Actinomadura*, *Glycomyces*, *Nonomuraea*, *Rhodanobacter*, *Luteibacter*, *Kribbella*, and *Streptomyces* (Fig. 4). The relative abundances of *Lysobacter*, *Devosia*, *Actinomadura*, *Glycomyces*, and *Nonomuraea* were higher in NPKM soil than in NPK soil, whereas the relative abundances of *Rhodanobacter*, *Luteibacter*, *Kribbella*, and *Streptomyces* showed the opposite trend.

**FIG 5:**
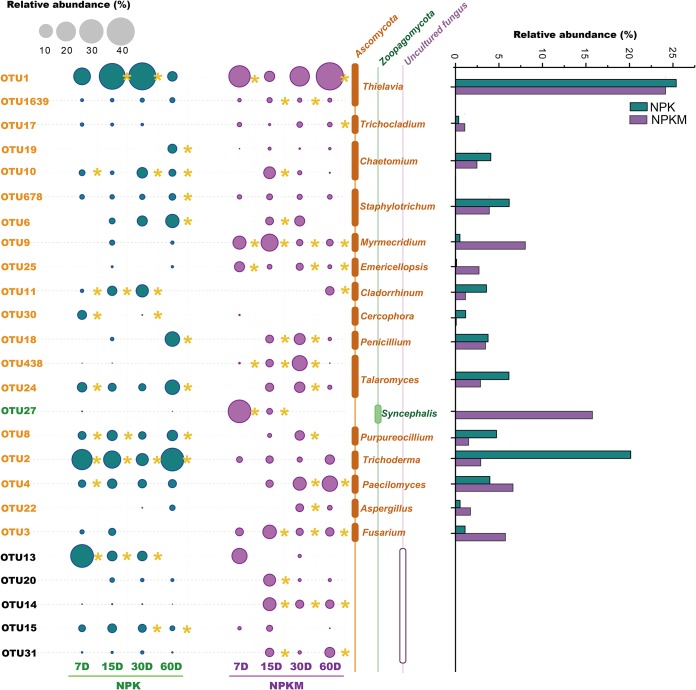
Dominant OTUs (relative abundance higher than 1%) in the fungal communities involved in the C flow from rice residues. The size of the circle represents the relative abundance of the OTU. The color of the circle represents the fertilization treatment, as indicated. Sampling was performed on the indicated days (D) after the addition of the rice residues. The yellow asterisk (*) to right of a circle indicates that the relative abundance with the respective treatment was higher than that with the other treatment. Taxa are indicated to the right of the plot. The histogram in the right panel represents the average relative abundances of the dominant genera at the four sampling time points.

The dominant OTUs of fungi in the NPK and NPKM soils were *Ascomycota* and *Zoopagomycotina*, and most were classified into the genera *Aspergillus*, *Thielavia*, *Trichocladium*, *Chaetomium*, *Emericellopsis*, *Cladorrhinum*, *Cercophora*, *Purpureocillium*, *Trichoderma*, *Fusarium*, and *Myrmecridium* (Fig. 5). Other identified OTUs were classified into the genera *Penicillium*, *Aspergillus*, *Talaromyces*, and *Staphylotrichum* of the *Eurotiomycetes*. OTU27, identified on day 7 as accounting for approximately 29.6% of the total labeled fungal sequences in NPKM soil, was identified as a member of *Syncephalis*; this OTU was entirely absent from NPK soils. Compared to levels with NPK, NPKM decreased the relative abundances of *Trichoderma*, *Purpureocillium*, *Talaromyces*, *Cercophora*, *Cladorrhinum*, *Chaetomium*, *Staphylotrichum*, and *Thielavia* and increased the relative abundances of *Fusarium*, *Aspergillus*, *Paecilomyces*, *Emericellopsis*, *Myrmecridium*, *Trichocladium*, and *Syncephalis*.

### Microbial networks involved in the C flow from rice residues.

**FIG 6:**
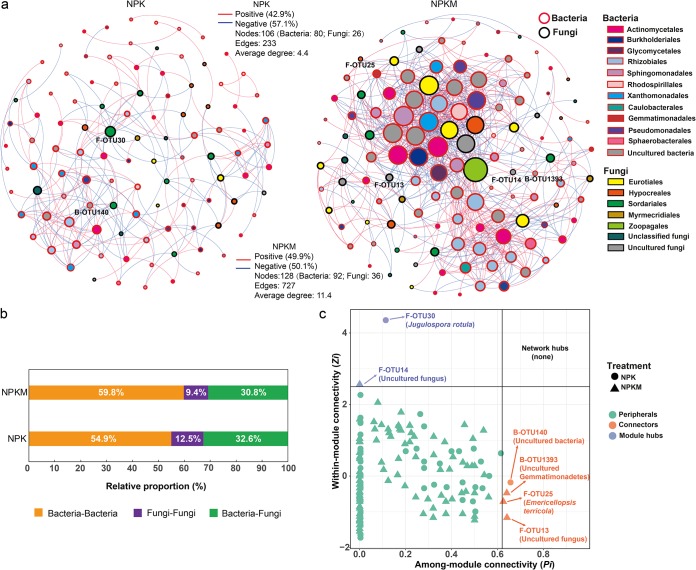
Molecular ecology network analyses of microbes involved in the C flow from rice residues in soils in relation to fertilization regimes. Labeled OTUs observed at four sampling time points from each fertilization treatment were pooled to construct networks that represent random matrix theory cooccurrence models. (a) Networks in soils treated with NPK or NPKM. The circles represent nodes. Circles with red edges represent bacterial (B) OTUs, whereas circles with black edges represent fungal (F) OTUs. Lines connecting two nodes represent the significant correlations between OTUs. Red lines represent significant positive correlations, and blue lines represent significant negative correlations. (b) Proportion of intrakingdom edges (edges between bacterial OTU and bacterial OTU or between fungal OTU and fungal OTU) and interkingdom edges (edges between bacterial OTU and fungal OTU) in the networks of NPK- and NPKM-fertilized soils. (c) The distribution of Zi and Pi values for the OTUs established from their module-based topological roles. The threshold values of Zi and Pi for categorizing OTUs were 2.5 and 0.62, respectively (74, 99). Circles, NPK treatment; triangles, NPKM treatment.

The microbes that played key roles in the flow of residue C were explored using microbial molecular ecology networks (Fig. 6). The bacterial and fungal OTUs with average relative abundances higher than 0.1% were selected for network construction by random matrix theory (RMT). There were 106 nodes in the NPK soil network, including 80 bacterial nodes and 26 fungal nodes. There were 128 nodes in the NPKM soil network, including 92 bacterial nodes and 36 fungal nodes. The average degree of the NPK network was 4.40, whereas that of the NPKM network was 11.4 (Table S2), indicating that the 3-year manure application increased the complexity of the microbial network related to the utilization of residue-derived C.

Two key network nodes were found in the NPK soil (Fig. 6 and Table S3): the fungal OTU30, affiliated with *Ascomycota*, acted as a module hub, and uncultured bacteria, OTU140, acted as an important connector between modules. Four key network nodes were present in the NPKM soil, including a module hub and three connectors. Specifically, the fungal OTU25 (*Ascomycota*), unclassified fungus (OTU13), and the bacterial OTU1393 affiliated with *Gemmatimonadetes* acted as connectors in the network; a module hub was found to be the unclassified fungus OTU14. The average relative abundances of the two fungal OTUs that served as module hubs in NPK and NPKM soils were 2.3% and 5.81%, respectively (Table S3). However, the average relative abundances of bacterial module connectors in both NPK and NPKM soils were less than 1%. The fungal OTU30 that acted as the module hub in NPK soil was identified as *Jugulospora rotula*, and the fungal OTU25 that acted as a connector in the NPKM soil was classified as *Emericellopsis terricola*.

### Response strategies of dominant microbes to residue amendment.

**FIG 7:**
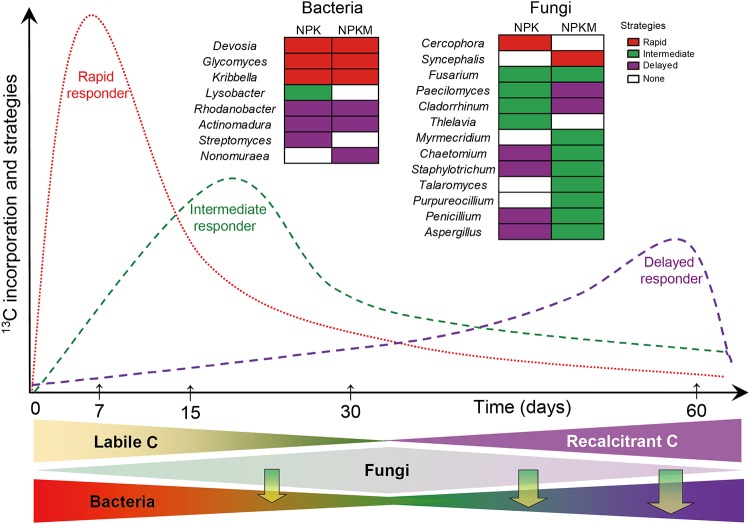
Conceptual diagram of microbial groups responsible for utilization and decomposition of rice residues in soils in relation to fertilization regimes. NPK- mineral fertilizers, NPKM- mineral fertilizers combined with manure. Line colors correspond to the colors assigned to the response strategies for rice residue utilization, as indicated on the right of the figure. Rapid responders, genera that displayed highest relative abundance at the outset (7 days after residue addition); intermediate responders, genera that displayed highest relative abundance at intermediate sample time points (between 15 and 30 days after residue addition); delayed responders, genera that displayed highest relative abundance at 60 days after residue addition. The lower part of the figure reflects C availability and the predominance of microbial groups. The vertical arrows represent the interactions between microbial groups in residue utilization (e.g., some carbon resources are provided by fungi to bacteria). Over time, the proportion of labile C in rice residue decreases while the proportion of recalcitrant C increases gradually. Most bacteria have a limited capacity to decompose recalcitrant components at later time points. Intermediate or delayed fungal responders can provide bacteria with resources that they cannot directly make from residues themselves, such as intermediate decomposition products (see text for details).

In order to assess the temporal dynamics of the microorganisms that use the residue-derived C, the abundant genera (Fig. 3 and 4) having significant changes between any two sampling points were selected. We assumed that the relative abundance changes between sampling points reflect microbial activities. Response strategies were assigned according to the time point at which the relative abundance of the genus was maximal. Genera that displayed highest relative abundances at the outset (7 days after residue addition) were referred to as rapid responders; those that displayed highest relative abundances at intermediate sampling time points (between 15 days and 30 days) were referred to as intermediate responders; those that displayed highest relative abundances at 60 days after residue addition were referred to as delayed responders. In NPK- and NPKM-fertilized soils, the response rates of the genera *Devosia*, *Glycomyces*, and *Kribbella* were the highest, whereas those of *Rhodanobacter* and *Actinomadura* were much lower, as were those of *Streptomyces* in NPK soil and *Nonomuraea* in NPKM soil. In contrast to the similar response strategies of bacteria in NPK and NPKM soils, fungi behaved quite diversely (Fig. 7). Only one genus, *Fusarium*, had the same response rates in the NPK and NPKM soils. In NPK soil, the genus *Cercophora* was the most rapid to respond to the addition of residue; *Thielavia*, *Cladorrhinum*, and *Paecilomyces* had lower response rates, and *Staphylotrichum*, *Chaetomium*, *Penicillium*, and *Aspergillus*, the delayed responders, were the slowest. In NPKM soil, *Syncephalis* responded rapidly while *Myrmecridium*, *Chaetomium*, *Staphylotrichum*, *Talaromyces*, *Purpureocillium*, *Penicillium*, and *Aspergillus* responded at intermediate levels, and *Paecilomyces* and *Cladorrhinum* exhibited delayed responses.

## DISCUSSION

### Fertilization changed the composition of microorganisms utilizing rice residues.

Fertilization regimes significantly changed the microbial community composition involved in rice residue assimilation (Fig. 3 to 5). The changes in the composition of the residue-assimilating microbial community are affected by the differences in nutrient content between NPK and NPKM soils (28, 46) as well as by organic matter availability. The modification of the microbial community by fertilization can be direct (addition of microorganisms living in manure) (47) and indirect, including the following methods: (i) by improving the pH buffering capacity through manure application (48, 49), (ii) by addition of diverse available organic compounds or nutrients with manure, (iii) by modification of C:N:P ratios, resulting in a change in the stoichiometric ratio of food resources for microorganisms (50, 51), and (iv) by increasing and stabilizing soil aggregates, resulting in modification of microbial habitats (40, 52).

In both NPK and NPKM soils, the rice residue facilitated the growth of specific microbial groups, such as members of the *Proteobacteria*, *Actinobacteria*, and *Ascomycota* (Fig. 1 and 2). The bacterial genera *Lysobacter* and *Streptomyces*, which have been described to be involved in the breakdown of potato tissue, wheat straw, rice straw, and corn cellulose (26, 29, 53, 54), are also the dominant bacteria utilizing the rice residue (Fig. 4). *Streptomyces* is commonly used in industrial applications to produce laccase and xylanase, using rice or wheat straw as substrates (55, 56). Microbial laccase production is usually related to the ability of the microbe to degrade lignin (57), and lignin is also the most difficult decomposable straw component. The higher abundance of *Streptomyces* in NPK than in NPKM soil may be related to the higher content of recalcitrant components in NPK soil resulting from the 3-year application of mineral-only fertilizers (58).

Most of the fungal groups were associated with the breakdown of cellulose (Fig. 5) as they can produce a variety of cellulolytic enzymes. Fungal genera *Trichocladium*, *Thielavia*, *Chaetomium*, and *Aspergillus* can produce cellulase or xylanase (59–63). *Chaetomium* and *Fusarium* have been identified in previous SIP studies as fungal genera that can utilize crop straw or cellulose (21, 22, 64). The application of manure increased the relative abundance of *Syncephalis*, whereas the relative abundance of *Trichoderma* was reduced. *Trichoderma* possesses a well-developed cellulase system containing many endoglucanases and two exoglucanases (65, 66). Various organic substances, such as monosaccharides, polysaccharides, and chitin have been shown to induce enzyme secretion (67), which may indicate that *Trichoderma* is more competitive for rice residue than other genera. *Trichoderma* has a capacity to mobilize and take up nutrients superior to that of many other soil microbes, especially under oligotrophic conditions, making it more efficient and competitive (68). Thus, the manure application in NPKM soil during the 3 years reduced *Trichoderma* development.

### Fertilization changed the cooccurrence patterns of microbes driving C flow from rice residues.

Network analyses provide a tool for identifying cooccurrence patterns and reflecting community organization (69–71) of microbes involved in the ^13^C-labeled residue C flow. Most previous studies have explored microbial molecular ecological networks in individual kingdoms, such as only fungi or only bacteria, but few showed the cooccurrence patterns between these groups. As microbial groups are not existing in isolation but potentially share niches in a given environment, network analyses were thus conducted on both bacteria and fungi (Fig. 6a). Linkages in microbial molecular networks can represent covariation between microbes or niche sharing (72). As our analysis might not have enough data points to construct a reliable network at each sample time, the network analyses were based on the combination of the samples across all sampling times for each fertilization regime. Pooling four sampling time points from each fertilization treatment in the network analysis can not only increase sensitivity for cooccurrence events but also show the cooccurrence patterns of microbes driving C flow from rice residues across the time points in each treated soil. Even though the microbial network will also be changed because of cross-feeding, the cross-feeding (microbes feeding on labeled microbial residues or microbial metabolites) also represents a type of C flow from rice residue through the microbial community. It is likely that the ^13^C label was turned over as the experiment progressed and that a second generation of microbial cells became labeled not only from ^13^C-labeled rice residue but also from ^13^C-labeled microbial metabolites, residues, and necromass of r-strategists that were formed earlier. Hence, it follows that our DNA-SIP data enabled the probing of C flow from rice residue into microbial communities in soils. Consequently, species that tended to negative correlations in the networks (Fig. 6a) indicate potential reuse of the microbial residue by some other microbes or potential competition in using rice residue. Conversely, any two species with a positive correlation reflect the potential cooperative or syntrophic in residue C utilization (Fig. 6a).

The network in NPKM soil was much more complex than that in NPK soil, with higher numbers of nodes, edges, average clustering coefficient, and average degree of the cooccurrences (Fig. 6a; see also Table S2 in the supplemental material). The complexity the of network is related to the changes in environmental factors, such as soil pH and C availability (31). An increased C input increases the functional complexity of soil microbial networks (31, 73). Three-year manure application fosters greater intermicrobial correlation and/or establishes more diverse shared niches, which represents a fundamental difference between NPKM and NPK soil. Keystone taxa acting as hubs or connectors in the network play critical roles in maintaining a network structure for other taxa (74). Fungal species, *Jugulospora rotula* in the NPK soil and *Emericellopsis terricola* in NPKM soil, were identified as keystone species (Fig. 6c; Table S3). *Emericellopsis terricola* was proved to have strong cellulase, protease, and laminarinase activities (75), which might be an essential factor for organizing microorganisms to cooperate in residue C utilization and may lead to a positive effect on the residue decomposition in NPKM soil. These keystone species, which are recognized as initiating components in networks, can also selectively alter microbial composition (76). Identifying these keystone groups is critical for predictive understanding of the subsequent potential for microbially mediated C sequestration when rice residue is returned to the soil. To better understand the role of these organisms in cooccurrence networks, uncultured keystone species should be the focus of future work.

Bacteria and fungi connected to each other in the network, and the organic amendment changed the ratio of the edges between bacteria and fungi to the total edges (Fig. 6b). In general, recalcitrant organic compounds in the residues (such as lignin and cellulose) are degraded mainly by fungi, and the released water-soluble substances can be utilized also by bacteria (77). Thus, fungi provide bacteria with resources that they cannot directly make from residues themselves, such as intermediate decomposition products. The 3-year input of manure increased C sources and their availability in the soil, including some soluble organics, which reduced the dependence of bacteria on fungi (78). In the NPK soil, which did not receive manure during the 3 years, bacteria strengthened their connection with fungi to get more soluble organics from residue decomposition. Thus, compared to the ratio in the NPK soil, NPKM fertilization decreased the ratio of edges between fungi and bacteria to the total edges (Fig. 6b). Therefore, the results provide some evidence that the cooccurrence patterns between the microbes are impacted by 3-year manure amendment. However, network cooccurrence patterns are ultimately based on statistical correlations, and they must be interpreted with caution as correlation does not directly demonstrate real microbial interactions. More solid experimental evidence, including more reasonable inference methods or promising culturing approaches, is still needed to explore complex ecological relationships under natural conditions and assess the effect of keystone species.

### Response strategies of microbes by utilization of rice residues depend on fertilization.

Plant residues contain complex organic compounds, such as water-soluble substances (low-molecular-weight organic substrates), polymeric carbohydrates (hemicellulose and cellulose), lignin, and phenolics (79–81). Each component can be decomposed or utilized mainly by specific taxonomic groups of microorganisms. Thus, rice residue degradation could be linked to a continuous change of microbial beta diversity over time (Fig. 3). The dynamics of microbial community structure was often related to the chemical composition of straw (82, 83). Decomposition of easily degradable components can stimulate the growth of early r-strategists (84), and as the availability of resources decreases, the relative abundances of microorganisms within this group will gradually decrease, while the late K-strategist groups will gradually come to dominate (85). Thus, decomposition of labile components during the first week leads to the rapid growth of certain groups of fungi and bacteria, such as the bacterial genera *Devosia*, *Glycomyces*, and *Kribbella*, and then the relative abundances of these groups decrease as the labile organic components are gradually consumed (Fig. 7). The relative abundances of *Rhodanobacter*, *Actinomadura*, *Streptomyces*, and *Nonomuraea* increased gradually as these bacteria could decompose recalcitrant components at later time points (Fig. 7). However, since the classification of the response strategies was at the genus level, little can be known about whether these members were involved directly in rice residue degradation or only indirectly by cross-feeding on the intermediate or metabolic products and necromass of the r-strategists.

Bacterial groups had similar response strategies in the two fertilized soils by assimilation of rice residue-derived C. The pure-culture methods have shown that bacteria could not grow on cellulose or crop residues unless they were cultured together with fungi (86). Thus, bacteria rely on fungi and their oxidative enzymes when substrates that are difficult to decompose are used. Consequently, the oxidative exoenzymes secreted by fungi [e.g., (per)oxidases] degrade the recalcitrant components into low-molecular-weight organic substrates that can be further utilized by bacteria (Fig. 7). Thus, no significant differences in the response strategies of the bacteria were found between the two fertilization regimes. However, the response strategies of the fungal genera were quite different between NPK and NPKM soils, which may be due to changes in the composition of the rice residue during the degradation (86).

### Conclusions.

The DNA-SIP approach enabled the identification of the key bacterial and fungal taxa of microbes involved in rice residue decomposition and utilization in soils under NPK and NPKM fertilization. The C from the rice residues was predominantly incorporated into bacterial phyla of *Proteobacteria* and *Actinobacteria* and the fungal phylum *Ascomycota*. Fertilization regimes had strong effects on microbial community composition, microbial response strategies, and microbial interactions involved in the C flow from rice residues. Since complete residue degradation involves multiple enzymatic steps that are conducted by many community members, our community-based analyses detected the microbial groups using the residues as well as the secondary products (metabolites) of initial residue cleavage. Bacterial response strategies for assimilating residues were independent of fertilization regimes. However, most of the fungal genera in soil receiving manure responded faster to residue addition than those in soil receiving solely mineral fertilizers. This is connected with the changes in the composition of the rice residue during degradation and with fungal adaptation (abundance and activity) to continuous manure input. This is confirmed by the more complex microbial network involved in the flow of rice residue-derived C in NPKM than that in NPK soils. Presumably, fungi release enzymes decomposing recalcitrant organic compounds in the residues (e.g., cellulose and lignin), and bacteria subsequently use the available soluble compounds produced by fungal exoenzymes. Future studies should be designed to discriminate the cross-feeding C flow between the species to understand mechanistic interactions of soil microbes that are directly and indirectly involved in residue-derived C utilization at any time point.

## MATERIALS AND METHODS

### Rice residue labeling.

Rice seeds (Oryza sativa cv. Zhendao11) were sterilized by exposure to 30% hydrogen peroxide for 30 min. Following a washing step, the rice seeds were cultured in the dark until germination. The seedlings were transferred into a transparent air-tight chamber and grown under hydroponic conditions with Hogland nutrient solution. The chamber was continually injected with ^13^CO_2_ (99 atom%; Cambridge Isotope Laboratories, Inc.) at a concentration of 400 μl liter^−1^. Additionally, before ^13^CO_2_ injection, high-purity air, which contained N_2_ (70%, vol/vol) and O_2_ (30%, vol/vol), was used to flush the chamber to remove the ^12^CO_2_. The environmental parameters of the illumination incubator were set as a 12-h photoperiod, temperature of 30°C day/20°C night, and relative humidity of 70% day/80% night. After 30 days of labeling, the top parts of the rice seedlings (including leaves and stems) were harvested and had a ^13^C content of 46 to 48 atom%. The rice residue was oven dried to constant weight at 65°C for further use.

### Soil microcosm setup.

Soils used for this study were collected from an experimental farm in Rugao, Jiangsu, China (120°51′E, 32°00′N). Rice (Oryza sativa cv. Zhendao11) was rotated annually with winter wheat (Triticum aestivum cv. Yangmai16). Soil samples were collected from a short-term (3-year) experimental site that included treatment with only mineral fertilizers (NPK) and treatment with mineral fertilizers plus manure (NPKM). The NPK-treated soil annually received 440 kg N ha^−1^ applied as urea (240 kg N ha^−1^ for the rice season and 200 kg N ha^−1^ for the wheat season), whereas the NPKM-treated soil similarly received a total of 440 kg N ha^−1^ in both seasons, of which 80% was from urea and 20% was from 7.41 tons ha^−1^ compost of pig manure. Both the NPK and NPKM treatments were applied annually with 128 kg P ha^−1^ and 116 kg K ha^−1^ as calcium superphosphate and potassium chloride, respectively. Each treatment was applied to three plots, which served as three replicates. Soils were collected from the top 0 to 20 cm after rice harvest in October 2015. Three soil samples for each fertilization treatment were obtained from the corresponding plots. Each soil sample consisted of 10 soil cores that were then sieved through 2-mm-pore-size sieves. The NPK-treated soil had a pH of 6.34, 20.34 g SOM kg^−1^, 1.22 g total N kg^−1^, 12.97 mg Olsen-P kg^−1^, and 92.00 mg NH_4_OAc-K kg^−1^; the NPKM-treated soil had a pH of 6.68, 22.52 g SOM kg^−1^, 1.38 g total N kg^−1^, 29.36 mg Olsen-P kg^−1^, and 85.67 mg NH_4_OAc-K kg^−1^.

Each soil sample was subjected to three microcosm treatments, including a negative control (soil without rice residue), positive control (soil with ^12^C-labeled rice residue), and ^13^C treatment (soil with ^13^C-labeled rice residue), and each microcosm treatment group contained six replicates. Each replicate comprised a microcosm of 10 g (oven-dried basis) of soil in a 125-ml hermetically sealed serum bottle. In total, 36 microcosms [(2 fertilization treatments × 3 replicates) × (3 microcosm treatments × 2 replicates)] were employed in the incubation experiment. All microcosms were subjected to a 14-day preincubation with soil water content adjusted to 60% of the soil maximum water-holding capacity and with an incubation temperature of 25°C before the beginning of the experiments. This preincubation adjusted all of the treatments to the environmental conditions used in the incubation stage to make the rice residue addition the unique variable in the microcosm incubations. After preincubation, 0.05 g of ^12^C-labeled rice residue or ^13^C-labeled rice residue (5 mg g^−1^ dws) was thoroughly amended into the positive control or ^13^C-treatment soils, respectively. For each microcosm treatment, one was used for the measurement of microbial respiration, and the remaining one was used for sampling for DNA isolation. Sterile water was supplied to each microcosm to adjust the soil moisture content to 60% of the soil maximum water-holding capacity. All soil microcosms were incubated at 25°C in the dark for 60 days, with daily adjustment of the soil water content. Soil samples used for DNA extraction were collected after 7, 15, 30, and 60 days of incubation.

### Measurements of soil respiration.

The measurement of microbial respiration was conducted at days 1, 7, 15, 30, and 60 after the addition of rice residue. The vials were closed with an air-tight butyl rubber stopper and incubated for 12 h at 25°C. Afterwards, the headspace CO_2_ concentration was analyzed by a gas chromatograph with a thermal conductivity detector operating at 60°C (Agilent 7890; Agilent, Santa Clara, CA). Separation was performed using a 177/149-mm (80/100 mesh) Chromosorb 102 column (Advanced Minerals, Santa Barbara, CA) at 40°C. The temperature of the injecting port was 100°C. The carrier gas (H_2_) flow rate was 80 ml min^−1^.

### DNA extraction and gradient centrifugation.

Soil DNA was extracted by a FastDNA spin kit for soil (MP Biomedicals, Cleveland, OH) according to the manufacturer’s protocol. A NanoDrop ND-2000 UV-visible light (UV-Vis) spectrophotometer (Thermo Scientific, Wilmington, DE) was used to measure the concentration and quality of extracted soil DNA. DNA density gradient centrifugation and fractionation were performed according to the methods of Kong et al. (87). Briefly, 3 μg of DNA was added into 1.85 g ml^−1^ of CsCl gradient buffer (0.1 M Tris-HCl, 0.1 M KCl, 1 mM EDTA, pH 8.0) with an initial CsCl buoyant density of 1.725 g ml^−1^, which was prepared by adjusting the refractive index to 1.4025 with an AR200 digital hand-held refractometer (Reichert, Inc., Buffalo, NY). Density gradient centrifugation was performed in a 5.1-ml Quick-Seal polyallomer ultracentrifugation tube (Beckman Coulter, Palo Alto, CA) in a VTi 65.2 rotor (Beckman Coulter), which was subjected to centrifugation at 177,000 × *g* (45,000 rpm) for 44 h at 20°C (Optima-XPN-100; Beckman Coulter, USA). Centrifuged gradients were fractionated into 14 equal volumes (∼340 μl) by displacing the gradient medium with sterile water at the top of the tube using a syringe pump ( pump LSP01-1A; Longer, China). Then, 50-μl aliquots were used to measure the refractive index to determine the buoyant density of each collected fraction. Fractionated DNA was precipitated from CsCl by addition of 500 μl of precipitating agent (30% polyethylene glycol [PEG] 6000 and 1.6 M NaCl), incubation for 1 h at 37°C, and then two washes with 70% ethanol. The samples were then dissolved in 30 μl of Tris-EDTA buffer.

### Real-time PCR quantification.

Quantitative real-time PCR was performed on an ABI 7500 real-time PCR system (Applied Biosystems, USA) to determine the abundances of bacterial 16S rRNA genes and fungal ITS1 using the primer pairs 338F/518R (338F, 5′-ACTCCTACGGGAGGCAGCAG-3′; 518R, 5′-ATTACCGCGGCTGCTGG-3′) (88) and ITS1/5.8s (ITS1, 5′-TCCGTAGGTGAACCTGCGG-3′; 5.8s, 5′-CGCTGCGTTCTTCATCG-3′) (89, 90), respectively, in the total soil DNA and fractionated DNA. Each reaction was performed in a total volume of 25 μl containing 12.5 μl of SYBR Premix Ex Taq (TaKaRa Biotechnology, Shiga, Japan), 0.5 μl of each primer (10 μM), 0.5 μl of ROX (6-carboxy-X-rhodamine) reference dye II (50×), 1 μl of DNA template (2 to 20 ng), and 10.5 μl of sterile water. Amplification conditions were as follows: 95°C for 5 min, 40 cycles of 15 s at 95°C and 34 s at 64°C, with a final temperature increase to 95°C for 15 s. Data were collected after each annealing step. One PCR product for each bacterial 16S rRNA gene and fungal ITS1 was gel purified using an Axygen PCR purification kit (Axygen Bio, USA), and the fragments were cloned separately into a pMD19-T vector (TaKaRa cloning kit). Then, ligated plasmid was transformed into competent DH5α Escherichia coli cells (TaKaRa). The reamplification and sequencing of white-positive clones using the vector-specific primers M13f/M13r were used to identify the correct insertion of the DNA fragments. Plasmids with correct inserts were extracted using an Axygen Plasmid Extraction kit (Axygen Bio, USA) for use as plasmid standards. A 10-fold dilution series of the plasmid standards was used to generate the standard curves. The PCR efficiency and correlation coefficient (*R*^2^) values for the standard curves were 95.68% and 0.9977, respectively, for 16S rRNA genes and 96.11% and 0.9978, respectively, for ITS1. The specificity of the amplification products was checked using melt curve analysis at the end of each PCR run and was then confirmed by standard agarose gel electrophoresis.

### High-throughput sequencing analysis.

Based on the relative abundance distribution patterns of the bacterial 16S rRNA genes and fungal ITS1 in 14 CsCl gradients, heavy fractions of the ^13^C-treatment samples and the corresponding fractions from the positive controls were chosen for sequencing. In total, 24 DNA samples (2 soils × 3 replicates × 4 sampling time points) were collected from the ^13^C-treatment heavy fractions, and 24 DNA samples (2 soils × 3 replicates × 4 sampling time points) were collected from the corresponding positive-control fractions. Sequencing of the V4-V5 regions from bacterial 16S rRNA genes (91) and of the fungal ITS1 (92) was conducted using an Illumina MiSeq System (Roche, Switzerland) at the Nanjing Genepioneer company (Nanjing, China). The raw data were processed using the Quantitative Insights into Microbial Ecology (QIIME) toolkit (93) and UPARSE standard pipeline (94). Reads with a quality score of less than 20 and length of less than 220 bp were discarded. Paired reads were merged using the fastq_mergepairs module of USEARCH (94, 95); pairs with mismatches in the overlap were eliminated, and only sequences of >220 bp in length were included in subsequent analyses. Then, singletons were discarded using UPARSE. In addition, the SILVA reference database was used to identify and remove putative chimeras. Taxonomic assignment was accomplished by clustering the sequences into operational taxonomic units (OTUs) at a 97% similarity level. Each sample was rarefied to the same number of reads (18,000 16S rRNA reads and 20,000 ITS1 reads) for downstream analyses using Mothur software. One representative sequence from each OTU was selected using the UPARSE pipeline, and in total 5,029 bacterial OTUs and 1,864 fungal OTUs were obtained in the OTU table (see Table S4 in the supplemental material). Each representative sequence was assigned to a Ribosomal Database Project (RDP) classifier for identification using a threshold of 0.8.

### Statistical analysis.

The OTUs involved in the decomposition and assimilation of ^13^C-labeled residue were filtered by comparing their relative abundances in the heavy fraction of ^13^C-treatment samples to those in the positive-control samples using R software with the DESeq2 package (96, 97). Unfortunately, the 16S rRNA sequencing data for one replicate in the NPKM positive control at 30 days was observed to be of much lower quality; thus, the mean of the NPKM positive control at 30 days was calculated based on the remaining two replicates for this analysis. Based on the result that the atomic percentage of ^13^C in the rice residue in the present study was lower than 50%, both the padj (false-discovery rate [FDR] adjusted *P* value) and the log_2_ fold change value were strictly controlled in subsequent analyses. The ^13^C-labeled OTUs were defined as the OTUs with padj values lower than 0.01 and log_2_ fold change values higher than 1. Nonmetric multidimensional scaling (NMDS) based on Bray-Curtis distances of all labeled OTUs and permutational multivariate analysis of variance (PERMANOVA) were performed using R (version 2.15.0) with the Vegan package. ^13^C-labeled OTUs with relative abundances higher than 1% were defined as the dominant OTUs involved in ^13^C-labeled residue decomposition and assimilation. Only genera that passed our filters for significant changes over time by analysis of variance (ANOVA) were included the assessment of response strategies for rice residue assimilation. Labeled OTUs observed in four sampling time points from each fertilization treatment were pooled to assess the roles of the microbes in driving the flow of rice residue-derived carbon using molecular ecological networks. The labeled OTUs with average relative abundances greater than 0.1% were selected to construct molecular ecological networks using the random matrix theory (RMT) (98), and the networks were visualized by Gephi (0.9.2). The threshold values of Zi (within-module connectivity) and Pi (among-module connectivity) for categorizing OTUs in networks are 2.5 and 0.62, respectively (74, 99). Furthermore, three categories that are organized as module hubs (Zi > 2.5), connectors (Pi > 0.62), and network hubs (Zi > 2.5 and Pi > 0.62) in the networks are defined as keystone microbes in the decomposition and assimilation of ^13^C-labeled rice residue. Origin, version 8.0, was used to draw the histogram; variance analysis was conducted using SPSS software (SPSS, version 16.0, for Windows; IBM Corp., Armonk, NY), and a *P *value of < 0.05 was defined as statistically significant.

### Data availability.

The sequences obtained in this study have been submitted to NCBI Sequence Read Archive (SRA) under the accession numbers PRJNA520803 and PRJNA518924.

## Supplementary Material

Supplemental file 1
