# Supplementary material for: DNA Stable-Isotope Probing Delineates Carbon Flows from Rice Residues into Soil Microbial Communities Depending on Fertilization
Source: Appl Environ Microbiol. 2020 Mar 18;86(7):e02151-19. doi: 10.1128/AEM.02151-19 (PMC7082572; doi:10.1128/AEM.02151-19)
Supplement: Supplemental file 1 [file AEM.02151-19-s0001.pdf]

Supplementary for:

DNA stable-isotope probing delineates carbon flows from rice residues into soil microbial communities depending on fertilization

Yali Kong [a], Yakov Kuzyakov [b,c], Yang Ruan [a], Junwei Zhang [a], Tingting Wang [a], Min Wang [a], Shiwei Guo [a], Qirong Shen [a], Ning Ling [a]*

a, Jiangsu Provincial Key Lab for Organic Solid Waste Utilization, Jiangsu Collaborative Innovation Center for Solid Organic Waste Resource Utilization, Nanjing Agricultural University, Nanjing, 210095, China.

b, Department of Soil Science of Temperate Ecosystems, Department of Agricultural Soil Science, University of Goettingen, Göttingen, Germany

c, Agro-Technology Institute, RUDN University, Moscow, Russia

*Corresponding author: Ning Ling

E-mail address: nling@njau.edu.cn; Tel. (+86) 25 84396853.

List of supplemental Tables

Table S1 Impact of various factors on the ^{13}C-labeled bacterial and fungal community across all samples.

Table S2 Topological properties of molecular ecology networks obtained for soils under different fertilization regimes.

NPK indicates soils with mineral-only fertilizers and NPKM indicates soil with mineral fertilizers combined with manure.

Table S3 Nodes identified as module hubs or connectors in the microbial networks involved in the flow of rice-residue-derived C in soils under different fertilization regimes.

NPK indicates soils with mineral-only fertilizers and NPKM indicates soil with mineral fertilizers combined with manure.

Table S4 Basic information of the bacterial and fungal operational taxonomic units (OTU) tables obtained in the heavy fraction after isopycnic gradient centrifugation.

NPK indicates soils with mineral-only fertilizers and NPKM indicates soil with mineral fertilizers combined with manure. The values 7D, 15D, 30D and 60D represent the sample time (day) after the addition of the rice residues. Data are means of three biological replicates.

Table S1 Impact of various factors on the ^{13}C-labeled bacterial and fungal community across all samples

	Bacteria			Fungi		
Factor	R^2	*P*	*F*	R^2	*P*	*F*
Treatments	0.52	0.001	159.9	0.29	0.001	290.4
Sample time	0.29	0.001	29.3	0.31	0.001	102.4
Treatments*Sample time	0.14	0.001	13.9	0.38	0.001	124.7

Adonis analysis was applied using package “vegan” from R, with permutation = 999.

Table S2 Topological properties of molecular ecology networks obtained for soils under different fertilization regimes.

Fertilization regimes	NPK	NPKM
Number of nodes	106	128
Total number of edges	233	727
Number of positive correlations	100	363
Number of negative correlations	133	364
Average path length	4.708	2.894
Average clustering coefficient	0.375	0.498
Modularity	0.583	0.426
Average degree	4.396	11.359

NPK indicates soils with mineral-only fertilizers and NPKM indicates soil with mineral fertilizers combined with manure.

Table S3 Nodes identified as module hubs or connectors in the microbial networks involved in the flow of rice-residue-derived C in soils under different fertilization regimes.

OTUID	Role	Abundance (%)	Phyla	Affiliation
**NPK**				
F-OTU30	Module hubs	2.3	Ascomycota	Jugulospora rotula
B-OTU140	Connectors	0.26	Unclassified	Uncultured bacteria
**NPKM**				
F-OTU14	Module hubs	5.81	Unclassified	Uncultured fungus
F-OTU25	Connectors	2.7	Ascomycota	Emericellopsis terricola
F-OTU13	Connectors	6.91	Unclassified	Uncultured fungus
B-OTU1393	Connectors	0.15	Gemmatimonadetes	Uncultured Gemmatimonadetes

NPK indicates soils with mineral-only fertilizers and NPKM indicates soil with mineral fertilizers combined with manure.

Table S4 Basic information of the bacterial and fungal operational taxonomic unit (OTU) tables obtained in the heavy fraction after isopycnic gradient centrifugation.

			Bacteria					Fungi				
Fertilization	Sample time	Residue	Number of reads	Number of OTUs	Number of reads after rarefaction	Number of total OTUs after rarefaction	Number of OTUs after rarefaction	Number of reads	Number of OTUs	Number of reads after rarefaction	Number of total OTUs after rarefaction	Number of OTUs after rarefaction
NPK	7D	^{12}C-residue	22781	2758	18000	5029	2571	29992	437	20000	1864	416
		^{13}C-residue	20606	1708	18000	5029	1621	27067	224	20000	1864	204
	15D	^{12}C-residue	22289	3074	18000	5029	2926	27052	484	20000	1864	466
		^{13}C-residue	20221	1886	18000	5029	1801	27417	275	20000	1864	253
	30D	^{12}C-residue	22969	3031	18000	5029	2864	32056	592	20000	1864	568
		^{13}C-residue	19617	1756	18000	5029	1705	29999	260	20000	1864	231
	60D	^{12}C-residue	22872	3137	18000	5029	3004	31429	491	20000	1864	464
		^{13}C-residue	21298	1978	18000	5029	1869	30311	244	20000	1864	216
NPKM	7D	^{12}C-residue	20101	2931	18000	5029	2839	25462	376	20000	1864	363
		^{13}C-residue	20182	1657	18000	5029	1605	30343	215	20000	1864	194
	15D	^{12}C-residue	21208	3060	18000	5029	2936	27771	525	20000	1864	503
		^{13}C-residue	20221	1876	18000	5029	1805	27153	331	20000	1864	301
	30D	^{12}C-residue	24681	2301	18000	5029	2113	26255	371	20000	1864	357
		^{13}C-residue	22073	1811	18000	5029	1695	28573	264	20000	1864	246
	60D	^{12}C-residue	21964	3310	18000	5029	3136	29986	480	20000	1864	461
		^{13}C-residue	21518	1995	18000	5029	1881	27577	265	20000	1864	241

NPK indicates soils with mineral-only fertilizers and NPKM indicates soil with mineral fertilizers combined with manure. The values 7D, 15D, 30D and 60D represent the sample time (day) after the addition of the rice residues. Data are means of three biological replicates.

List of supplemental Figures

Fig. S1 Effects of rice residue addition on the efflux rate of CO_2 (a), the abundance of bacteria (b) and fungi (c), and the ratio of fungal to bacterial abundance (d) in soils under different fertilization regimes. The values 7D, 15D, 30D and 60D represent the sample time (day) after the addition of the rice residues. Data are means of three biological replicates. Different letters above the columns indicate significant differences between different treatments in each sampling point (P < 0.05). NPK indicates soils with mineral-only fertilizers and NPKM indicates soil with mineral fertilizers combined with manure.

Fig. S2 The relative abundance distribution of DNA from bacteria (a, b) and fungi (c, d) across the CsCl density gradient. Data are the means of three biological replicates that from day 17. NPK indicates soils with mineral-only fertilizers and NPKM indicates soil with mineral fertilizers combined with manure.

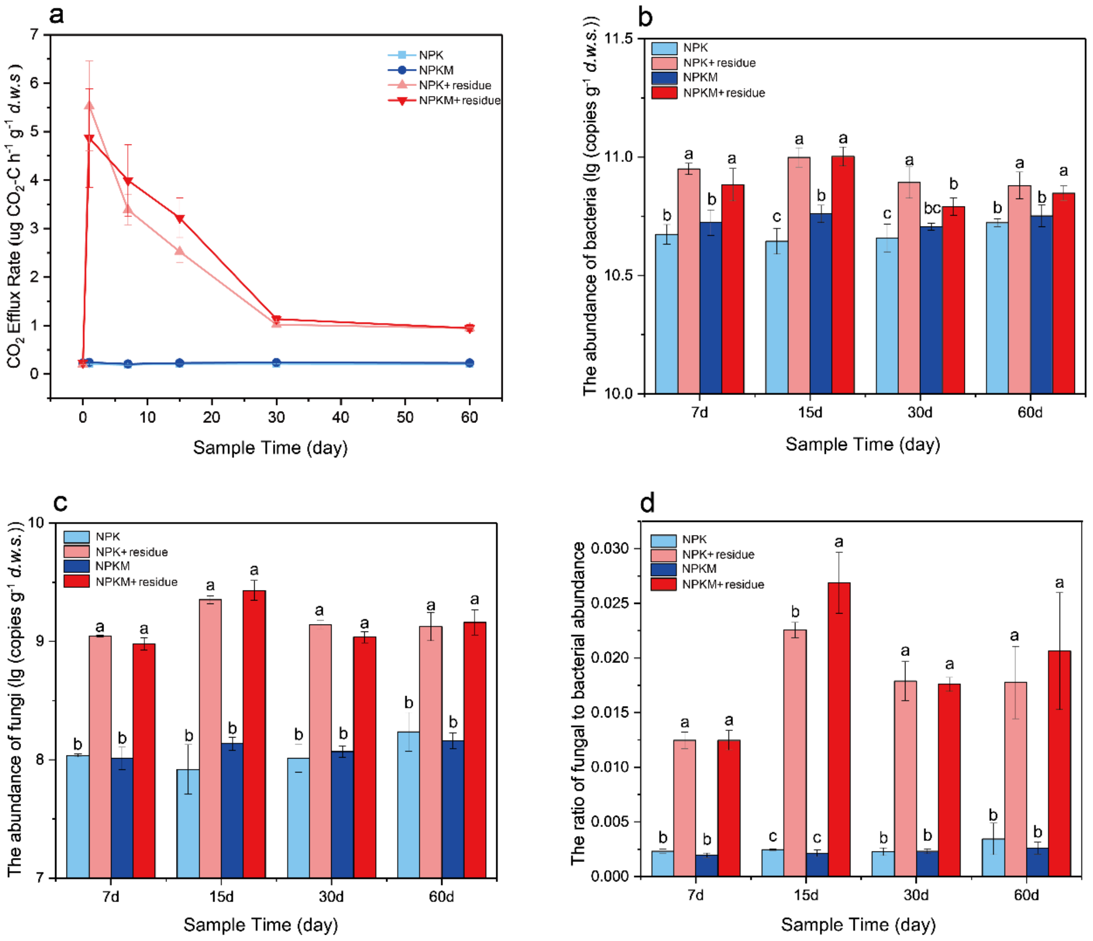

Fig. S1 Effects of rice residue addition on the efflux rate of CO_2 (a), the abundance of bacteria (b) and fungi (c), and the ratio of fungal to bacterial abundance (d) in soils under different fertilization regimes. The values 7D, 15D, 30D and 60D represent the sample time (day) after the addition of the rice residues. Data are means of three biological replicates. Different letters above the columns indicate significant differences between different treatments in each sampling point ($P < 0.05$). NPK indicates soils with mineral-only fertilizers and NPKM indicates soil with mineral fertilizers combined with manure.

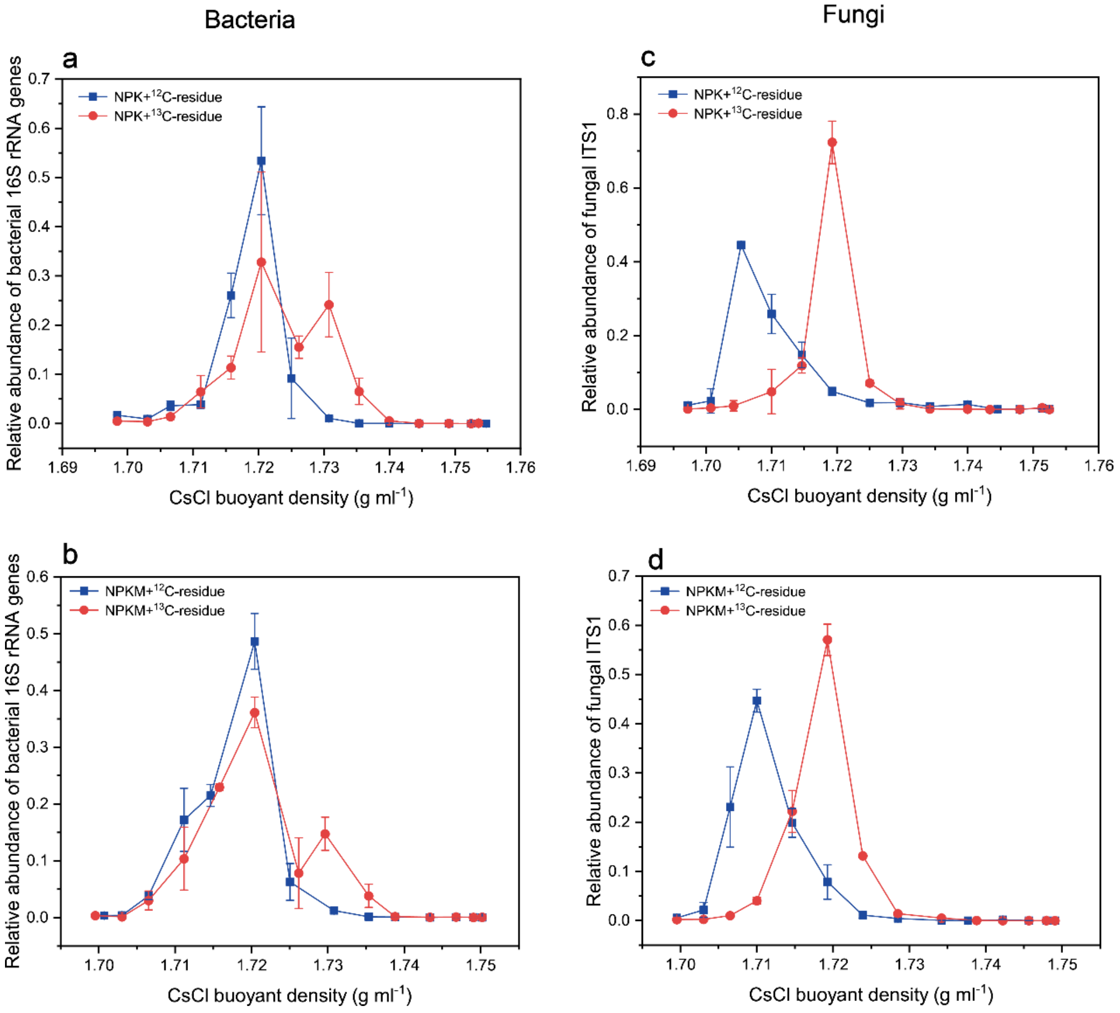

Fig. S2 The relative abundance distribution of DNA from bacteria (a, b) and fungi (c, d) across the CsCl density gradient. Data are the means of three biological replicates that from day 17. NPK indicates soils with mineral-only fertilizers and NPKM indicates soil with mineral fertilizers combined with manure.
